# A Path-Based Distribution Measure for Network Comparison

**DOI:** 10.3390/e22111287

**Published:** 2020-11-12

**Authors:** Bing Wang, Zhiwen Sun, Yuexing Han

**Affiliations:** 1 School of Computer Engineering and Science, Shanghai University, Shanghai 200444, China; sunzhiwen@shu.edu.cn; 2 Shanghai Institute for Advanced Communication and Data Science, Shanghai University, Shanghai 200444, China

**Keywords:** network comparison, path distribution, network entropy, network reduction

## Abstract

As network data increases, it is more common than ever for researchers to analyze a set of networks rather than a single network and measure the difference between networks by developing a number of network comparison methods. Network comparison is able to quantify dissimilarity between networks by comparing the structural topological difference of networks. Here, we propose a kind of measures for network comparison based on the shortest path distribution combined with node centrality, capturing the global topological difference with local features. Based on the characterized path distributions, we define and compare network distance between networks to measure how dissimilar the two networks are, and the network entropy to characterize a typical network system. We find that the network distance is able to discriminate networks generated by different models. Combining more information on end nodes along a path can further amplify the dissimilarity of networks. The network entropy is able to detect tipping points in the evolution of synthetic networks. Extensive numerical simulations reveal the effectivity of the proposed measure in network reduction of multilayer networks, and identification of typical system states in temporal networks as well.

## 1. Introduction

Network approach is a powerful tool to describe complex systems [1,2,3], since the basic unit in a complex system can be represented by node, and the interaction between nodes can be represented by edge. Identifying and quantifying the structural differences between networks is a very important and challenging problem in network science, and it can be applied to compare brain networks [4,5], detect sequences of system states in temporal networks [6], and even reduce multilayer networks [7].

Many methods have been proposed for network comparison, and are often described as similarity measures or distances between two networks [8,9]. Basic approaches usually measure how many nodes or edges the two graphs have in common, such as Hamming distance and Jaccard distance [10]. The former measures the number of edge deletions and insertions necessary to transform one graph into another, which is more sensitive to the density of the network; while the latter includes a normalization process with respect to the total number of edges in two networks. Both the Hamming distance and Jaccard distance tend to grossly oversimplify the problem and miss key information of the similarities and differences in the network topology, since they treat all the change of edges uniformly, and they can be seen as special instances of graph edit distance (GED) [11,12,13]. Furthermore, according to the eigenvalues of either the adjacency matrix or the Laplacian matrix of the network, researchers defined a class of measures based on spectral distance for network comparison [9,14,15]. Then, a more general measure extended the spectral distance with the power of eigenvalues, capturing network information from local to global scale, has been proposed [9]. Very recently, researchers propose a class of methods for network comparison by embedding the network into a low dimensional space [16]. For instance, Shrivastava et al. proposed a covariance matrix composed of normalized vectors of the adjacency matrix, describing the spectrum of the adjacency matrix and sub-structures in the network [17].

Recently, some studies took use of network topology information with information theory for network comparison [7,18,19,20,21]. For instance, according to the topological information of nodes and their neighbors in the network, Carpi et al. proposed a diversity measure of a system composed of several components, then identified similar network layers according to there contribution to a global diversity value [18]. With quantum theory, Domenico et al. treats two networks as two mixed states and then compare them with information theory [7]. Paths in the network usually describes the communication capability between nodes. The shorter the path is, the stronger the interaction between the nodes will be. Based on this observation, Schieber et al. proposed a method based on quantifying the difference between distance probability distributions, which can identify and quantify structural difference in networks that have a practical impact on the information flow through the network, such as the presence or absence of critical links that connect or disconnect connected components [19]. Wang et al. used the communicability between two nodes to define the communicability sequence entropy of networks, then use the Jensen-Shannon divergence as distance measure between two networks [20]. It not only can accurately quantify the structural dissimilarities between synthetic networks, but also be able to identify the critical percolation probability of the random network in the evolutionary process. Bagrow et al. introduced a new measure, named as network portrait, to compare networks. The portrait matrix describes the distance distribution, which is mathematically principled and incorporates the topological characteristics of networks at all structural scales [21].

Although path-based methods are able to capture characteristics of network topology on a global scale, the information they provide is still very limited or incomplete.Inspired by their work, here, we propose a new measure for network comparison by making use of the path information as well as node centrality, such as degree, capturing network features from local to global scale. Depending on the availability of the two end nodes’ information along a given path, we propose a path-based distribution, combined with one or two end nodes’ information, respectively, to characterize the network topology. Furthermore, we define the network entropy and network distance according to the path-based distributions. We find that, combining more end nodes’ information to the path distribution can further amplify the distance between two networks. We also apply our method to resolve network reduction of multilayer networks, which successfully aggregates layered networks. The applications of our measures in temporal networks are also able to identify typical states shown in the interaction patterns. In all, experiments on synthetic networks and real networks reveal the effectivity of our measures in identifying the critical percolation probability in network evolution, quantifying dissimilarity of two networks, reducing multilayer networks, and identifying typical system states in temporal networks.

The rest of this paper is organized as follows. In Section 2, we describe the basic path distribution, and combine the path distribution with end node’s information, based on which we further define the network entropy and network distance between two networks. Next, we apply our measures to both synthetic networks and real-world datasets in Section 3, demonstrating its effectiveness in characterizing network evolution, network distance of two networks, as well as network reduction and identification of typical status in temporal networks. Finally, we conclude and summarize our work in Section 4.

## 2. Methods

### 2.1. Path Distribution Combined with End Nodes’ Information

For a network *G* with *N* nodes, let $n_{l}\left(v_{i}\right)$ denote the number of nodes whose distance to $v_{i}$ is *l*, where $0\leq l\leq L_{max}$, and $L_{max}$ is the diameter of the network *G*. We define the distance between node *i* and *j* is zero if i=j. Accordingly, for a given node *i*, the number of nodes at distance 1 to node *i* is exactly the degree of node *i*. Similarly, the number of nodes at distance 2 to node *i* is equal to the number of triple structure from node *i*, and so on.

By using the distribution of nodes accounting for the given path length, a lot of information can be traced. For instance, Stella et al. [22] considered the distribution of nodes whose distance to a give node *i* is *l*, that is, $p_{i}\left(l\right)=\frac{n_{l}\left(v_{i}\right)}{\sum_{l=0}^{L_{max}}n_{l}\left(v_{i}\right)}$, to define distance entropy of a node in the network, given by $h_{i}=\sum_{l=0}^{l_{max}}p_{i}\left(l\right)\log p_{i}\left(l\right).$ Chen et al. [23] proposed the network entropy by focusing on a given path length *l*, where the ratio of nodes at distance-*l* to a given node is counted, described as

(1) $$h_{l}=-\sum_{i=1}^{N}\frac{n_{l}\left(v_{i}\right)}{\sum_{j=1}^{N}n_{l}\left(v_{j}\right)}\log\left(\frac{n_{l}\left(v_{i}\right)}{\sum_{j=1}^{N}n_{l}\left(v_{j}\right)}\right).$$

In the present work, instead of using the local information around a specific node or a fixed distance between nodes, here, we focus on the distribution of all pairs of nodes at a given distance *l*. Note that $n_{l}\left(v_{i}\right)$ encodes many structural features of the network. Obviously, $\sum_{i=1}^{N}n_{0}\left(v_{i}\right)=N$ stores the number of nodes in the graph, while $\sum_{i=1}^{N}n_{1}\left(v_{i}\right)$ is twice as much as the number of edges in networks. Similarly, $\frac{1}{2}\sum_{i=1}^{N}n_{2}\left(v_{i}\right)$ depicts the number of triple structures in the network.

Based on the above analysis, we propose a distribution characterized by distance *l*, p(l), that is, the number of pairs of nodes whose distance being *l* over the number of all pairs of the nodes at all the possible distances, given by

(2) $$p\left(l\right)=\frac{\sum_{i=1}^{N}n_{l}\left(v_{i}\right)}{\sum_{l=0}^{L_{max}}\sum_{i=1}^{N}n_{l}\left(v_{i}\right)}.$$

In other words, p(l) is the probability that a pair of randomly selected nodes is at distance *l*. Then, based on the path distribution, we can define the network entropy as the Shannon entropy [24], given by,

(3) $$H_{l}=-\sum_{l=0}^{L_{max}}p\left(l\right)\log p\left(l\right).$$

$H_{l}$ is able to characterize the path distribution in the network. A network with a high diversity of distance patterns would has a high value of $H_{l}$.

To demonstrate how the measure $H_{l}$ characterizes the network models, we consider several specific networks with size *N*. In complete networks, the distance between each pair of nodes is 1. Thus, the path distribution and the corresponding network entropy are given as follows:

(4) $$p\left(l\right)=\left\{p\left(0\right)=\frac{N}{N^{2}},p\left(1\right)=\frac{N(N-1)}{N^{2}}\right\},H_{l}=\log N-\frac{N-1}{N}\log\left(N-1\right)\to 0.$$

If the network size is sufficiently large, the network entropy $H_{l}$ will approach to 0.

In star networks, there exists a central node linked to all other nodes. The distance between peripheral nodes is 1, while the distance between peripheral nodes and the central node is 2. Then, the path distribution and the network entropy are calculated as:

(5) $$\begin{array}{cc} p(l) & =\left\{p\left(0\right)=\frac{N}{N^{2}},p\left(1\right)=\frac{2(N-1)}{N^{2}},p\left(2\right)=\frac{\left(N-1)(N-2\right)}{N^{2}}\right\},\\ H_{l} & =\frac{2N-1}{N}\log N-\frac{2(N-1)}{N^{2}}\log2\left(N-1\right)-\frac{\left(N-1)(N-2\right)}{N^{2}}\log\left(N-1\right)\left(N-2\right). \end{array}$$

Note that the path distribution of star networks is broader than complete networks, where most of the shortest path length is 2. Although $H_{l}$ for star networks is higher than that of complete network, $H_{l}\to 0$ when N→∞ in star networks.

In ring networks, each node connects to its left and right nodes, the diameter $L_{max}$ of ring networks is ⌊N/2⌋, where ⌊.⌋ is floor function. In ring networks the length of the shortest path between an arbitrary pair of node is uniformly distributed. If *N* is odd, then, the distribution of the shortest path is $p\left(l\right)=2N/N^{2}$ for l≠0, and $p\left(0\right)=N/N^{2}$. Consequently, the network entropy for ring networks is given by:

(6) $$H_{l}=\frac{1}{N}\log N+\frac{N-1}{N}\log\frac{N}{2}.$$

If *N* is even, $p\left(l\right)=2N/N^{2}$ for $l\neq 0,L_{max}$, and $p\left(0\right)=p\left(L_{max}\right)=N/N^{2}$. Then, the network entropy becomes:

(7) $$H_{l}=\frac{2}{N}\log N+\frac{N-2}{N}\log\frac{N}{2}.$$

In all, $H_{l}\approx \log N/2$. Notably, since ring network is a special case of ring lattice network, where each node has *K* neighbors (K=2 in ring network), the analytical results for ring network can also be extended to ring lattice network with K>2 as $H_{l}\approx \log N/K$. If K=N−1, the network becomes a complete network and $H_{l}\to 0$.

Note that p(l) measures the global distribution of paths with length *l* and $H_{l}$ depicts the property of distance between an arbitrary pair of nodes in the network while neglecting the end nodes’ properties along the path. A combination of the end nodes’ properties, such as degree, closeness centrality, betweenness centrality and eigenvector centrality [25,26], with the path distribution would further detail characteristics of the path. To further explore how the path distribution p(l) relates to the end nodes’ properties, we combine one of the end nodes’ centrality *c* into the distribution p(l) to define an extended path distribution, p(c,l), where p(c,l) is the probability that a pair of randomly selected nodes is at a distance *l* and one end node’s centrality is *c*. Moreover, since some centrality measures, such as degree, betweenness, and eigenvector centrality are positively correlated [27], here, for simplicity, a node’s degree *k* is borrowed into the path distribution being represented as p(k,l).

Let p(k,l) denote the fraction of a pair of nodes with distance *l* and one of the end nodes’ degree being *k*. The relationship between p(k,l) and p(l) is demonstrated by simple calculations. Obviously, p(k,l) is able to recover the shortest path distribution p(l) by summing up all the possible degree obtained at the end node, given by,

(8) $$\;p\left(l\right)=\sum_{k=0}^{K_{max}}p\left(k,l\right),$$

where $K_{max}$ is the maximum degree in network *G*. Besides, p(k,l) can also recover to the degree distribution p(k) by setting l=0, read as

(9) $$\;p\left(k\right)=\frac{p(k,0)}{\sum_{k=0}^{K_{max}}p\left(k,0\right)}.$$

The distribution of the nearest neighbors in the network is also captured with l=1. Similarly, $\frac{p(k,l)}{\sum_{k=0}^{K_{max}}p\left(k,l\right)}$ can also been taken as degree distribution of *l*-order neighboring nodes.

As mentioned above, the distribution p(k,l) incorporates one end node’s degree into the path distribution. To further explore how the end nodes’ properties affect the path distribution, it is natural to extend the above definition to consider two end nodes’ properties into the path distribution, that is, $p(k,k^{{}^{\prime }},l)$, which describes the probability that a pair of randomly selected nodes are at a distance *l* and the end nodes’ degree are *k* and $k^{\prime }$ respectively. Obviously, $p(k,k^{{}^{\prime }},l)$ captures all the information that p(k,l) contains, since $p\left(k,l\right)=\sum_{k^{{}^{\prime }}=0}^{K_{max}}p\left(k,k^{{}^{\prime }},l\right)$. Compared to the one end node path distribution p(k,l), $p(k,k^{{}^{\prime }},l)$ further details the characteristics of a path.

Based on the above analysis, we can define the network entropy with the above two extended path distributions, respectively. Since network entropy is scalar and can be used as a complexity measure to describe a network system [28,29], we apply the Shannon entropy with the probability distribution to serve as an index of the feature of the network structure. By combining one end node’s degree into the path distribution, p(k,l), the network entropy is defined as follows:

(10) $$H_{k,l}=-\sum_{k=0}^{K_{max}}\sum_{l=0}^{L_{max}}p\left(k,l\right)\log p\left(k,l\right).\;$$

Furthermore, by combining the two end nodes’ degree into the path distribution, $p(k,k^{\prime },l)$, we have

(11) $$H_{k,k^{\prime },l}=-\sum_{k^{{}^{\prime }}=0}^{K_{max}}\sum_{k=0}^{K_{max}}\sum_{l=0}^{L_{max}}p\left(k,k^{{}^{\prime }},l\right)\log p\left(k,k^{{}^{\prime }},l\right).$$

So far, the two extended network entropies are defined, that is, $H_{k,l}$ and $H_{k,k^{\prime },l}$, by combining the end node’s degree.

**Figure 1:**
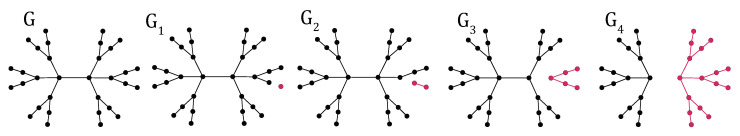
Schematic representation of five different networks with the same number of nodes. $G_{1}-G_{4}$ are obtained by deleting one edge from network *G*. $G_{1}$ has one isolated node; $G_{2}$ has one disconnected component with two nodes; $G_{3}$ has one disconnected component with five nodes; $G_{4}$ is disconnected into two balanced connected components.

Let us illustrate the path-based entropies with a simple example in Figure 1. There are totally 32 nodes in network *G*. By removing one edge from *G*, we obtain four networks denoted as the set {$G_{1}$, $G_{2}$, $G_{3}$, $G_{4}$}. As one edge is removed, depending on the choice of the removed edge, network *G* is separated into different components. For instance, $G_{1}$ is composed of two components with one isolated node, which is most similar to network *G*. While $G_{4}$ is composed of two balanced components and it would be the most different one from network *G*. We calculate the three network entropies, $H_{l}$, $H_{k,l}$, and $H_{k,k^{\prime },l}$ for each network, respectively. As Table 1 shows, after the one edge being removed, the shortest path distribution and the network entropy for each network has changed. For instance, when the critical edge that linked the two balanced components, is removed from $G_{4}$, and the network entropy has a significant change compared to *G*. $H_{k,k^{{}^{\prime }},l}$ describes the network with the most detailed information. As more nodes’ degree combined into the path, the path distribution is better discriminated, and the value of network entropy increases.

We have to remark that the proposed network entropy can also be applied to directed networks by counting directed paths as well as weighted networks by summing up all edge weights along a path. The network is not necessary to be connected, since the path can be counted in network components.

### 2.2. Network Distance between Two Networks

Based on the above proposed path distributions combined with end nodes’ properties, we can define the distance between two networks. Here, the Kullback–Leibler divergence ($D_{KL}$) is used to calculate the difference between two probability distributions,

(12) $$D_{KL}\left(p||q\right)=p\log\frac{p}{q},$$

where *p* and *q* are path-based distributions of network *P* and network *Q*. In this paper, p(l), p(k,l), and $p(k,k^{{}^{\prime }},l)$ are used to describe the characterized distribution of networks, respectively. Since the Kullback–Leibler divergence is not symmetric and does not define a distance, a more suitable quantity to measure the dissimilarity between two distributions is necessary. Let us set $\mu =\frac{\left(p+q\right)}{2}$, the Jensen-Shannon divergence ($D_{JS}$) is defined as:

(13) $$D_{JS}\left(p||q\right)=\frac{1}{2}(D_{KL}\left(p||\mu \right)+D_{KL}\left(q||\mu )\right)=H\left(\mu \right)-\frac{1}{2}\left(H\left(p\right)+H\left(q\right)\right),$$

where H(p)=−∑plogp, which is the Shannon entropy for distribution *p*. $D_{JS}$ is reflexive and symmetric. In addition, $\sqrt{D_{JS}}$ takes values in [0,1] and satisfies all the properties of a metric [30]. In the following, we deploy $d_{PQ}=\sqrt{D_{JS}}$ to quantify the distance between two distinct networks *P* and *Q*. We note that $d_{PQ}=0$ only if *P* and *Q* have same path-based distributions. $d_{PQ}=1$ if *p* and *q* are completely disjointed, which means p(x)≠0 and q(x)=0 for all *x*.

**Figure 2:**
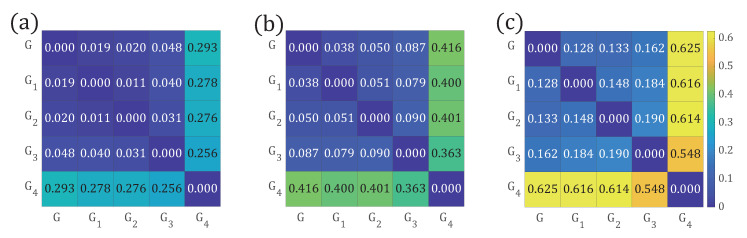
The distance matrices between networks presented in Figure 1 are calculated. (**a**) Distances between *G* and $\left\{G_{1},G_{2},G_{3},G_{4}\right\}$ are calculated with p(l); (**b**) Distances between *G* and $\left\{G_{1},G_{2},G_{3},G_{4}\right\}$ is calculated with p(k,l); (**c**) Distances between *G* and $\left\{G_{1},G_{2},G_{3},G_{4}G_{4}\right\}$ is calculated with $p(k,k^{{}^{\prime }},l)$.

Let us verify if the distance between the networks becomes larger with the proposed path distributions, p(l), p(k,l), and $p(k,k^{\prime },l)$. We calculate the $\sqrt{D_{JS}}$ between each pair of the five networks, respectively, and obtain the distance matrix in Figure 2. We see that the distance between *G* and $G_{4}$ is the largest for each path distribution in Figure 2a–c. The distances between $G_{4}$ and the remaining networks decrease in order. All the three measures show that $G_{1}$, $G_{2}$, and $G_{3}$ are more similar to *G* than $G_{4}$, as $G_{1}$, $G_{2}$, and $G_{3}$ have small disconnected components, which have less impact on information flow in the network. This result is reasonable and acceptable. Moreover, with the introduction of the end nodes’ degree in the path distribution, p(k,l) and $p(k,k^{\prime },l)$, we see that the distance between each pair of networks becomes larger (Figure 2b,c).

As another example, let us observe the distances between $G_{4}$ and $G_{1}$, and $G_{4}$ and $G_{2}$. Intuitively, the information flow in both $G_{2}$ and $G_{4}$ is blocked more than $G_{1}$. We see that the distances based on p(l) and $p(k,k^{\prime },l)$ correctly find the fact that $G_{4}$ is more similar to $G_{2}$ than $G_{1}$, while the distance calculated with p(k,l) does not. In addition, the distance value increases with the introduction of more detailed properties on the end nodes of the path, thus, networks can be more effectively discriminated.

Our proposed measure of network distance is grounded in information theory, which fully utilizes the structural topological information of the network and the divergence measure. Both undirected and directed networks can be treated naturally, and disconnected networks can also be handled without any specifications.

## 3. Results

In this section, we testify if the network entropy defined on the path distribution combined with end node’s degree can effectively identify percolation point in the network evolution. We also evaluate the performance of the network distance measure, calculated with different path distributions, for discriminating distinct network models. We also apply the network distance measure to network reduction of multilayer networks composed of synthetic networks as well as real data.

### 3.1. Experiments on Synthetic Networks

We build three types of network models usually considered in the literature, that is, Erdos–Renyi (ER) random graphs [31], Watts–Strogatz small-world (WS) networks [32], and Barabasi–Albert (BA) networks [33], respectively. Without specification, the network size is set as N=200.

#### 3.1.1. Comparison of Network Entropies Based on Different Path Distributions

Firstly, we testify if the network entropy calculated with different path distributions, $H_{l}$, $H_{k,l}$, and $H_{k,k^{\prime },l}$ can identify the emergence of the giant component in ER networks, and the presence of small world phenomena in WS networks, respectively. In order to achieve the goal, we generate ER networks with different connecting probability *p* and calculate the network entropy $H_{l}$, $H_{k,l}$, and $H_{k,k^{\prime },l}$, respectively, for each case. Similarly, we generate WS networks with different rewiring probability *r* and calculate the three network entropies in a similar way.

**Figure 3:**
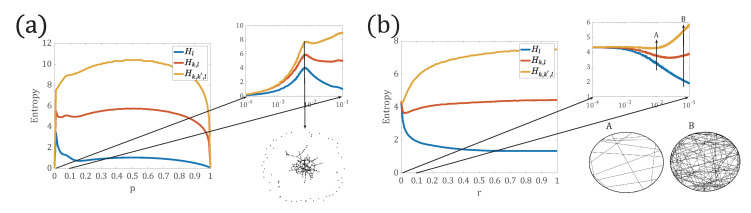
Comparison of network entropies defined on different path distributions. (**a**) Network entropies calculated for Erdos–Renyi (ER) networks with connecting probability *p*. The right panel highlights the critical connecting probability $p_{c}$ where the giant component emerges. (**b**) Network entropies calculated for WS networks with rewiring probability *r*. The right panel highlights the critical rewiring probability $r_{c}$ at which the “Small World" characteristics appears within the range of *A* to *B*.

From Figure 3a, we see that when *p* is small, most of the nodes in the network are isolated and there are very few paths between them, and thus, all the three entropies are very small. As *p* increases, at some critical value of p≈1/N, the three network entropies increase sharply, where the giant component of the network appears. After the emergence of the giant component, the addition of more edges shortens the distance between arbitrary pair of nodes, thus, p(l) is more narrowly distributed, so $H_{l}$ decreases. When the degree distribution becomes wider, the curve of $H_{k,l}$ reaches a plateau. The curve of $H_{k,k^{\prime },l}$ still rises until the degree distribution becomes narrow again. The more detailed end nodes’ degree of the path distribution is obtained, the higher the network entropy is, that is, $H_{k,k^{\prime },l}>H_{k,l}>H_{l}$. With further increase of *p* approaching to 1, the network almost becomes a complete graph, where the degree distribution and the path distribution are more homogeneous, thus, all the three network entropies are close to 0.

In a similar way, Figure 3b shows the network entropies measured for the three path distributions on WS networks with different rewire probability *r*. When r=0, the network is regular and all nodes’ degree are same with $\frac{\langle k\rangle }{2}$, while the path of each pair of nodes is diversely distributed, hence, $H_{l}$ achieves the maximum value. With more edges being rewired, the path distribution becomes more homogeneous, thus, $H_{l}$ decreases gradually. For $H_{k,l}$, we see that $H_{k,l}$ decreases slightly and then increases to a stable value. The decrease of the entropy $H_{k,l}$ is due to the appearance of small world characteristics by rewiring edges. With the rewiring process, the average path length of the network becomes less and the nodes’ degree is broadly distributed. Finally, for $H_{k,k^{\prime },l}$, with the rewiring of edges, the network becomes more irregular and nodes’ degrees are further widely distributed, thus, the network entropy $H_{k,k^{\prime },l}$ also increases to a high value.

In all, all the three network entropies are able to capture the features of evolving networks, such as the emergence of the giant component and the appearance of small world characteristics. With more detailed information on the end nodes of the path, the network entropy becomes larger.

#### 3.1.2. Comparison of Network Entropy for Network Models with Different 〈k〉

Next, we compare the three network entropies on three types of networks, that is, ER networks, WS networks, and BA networks for different average degree 〈k〉.

**Figure 4:**
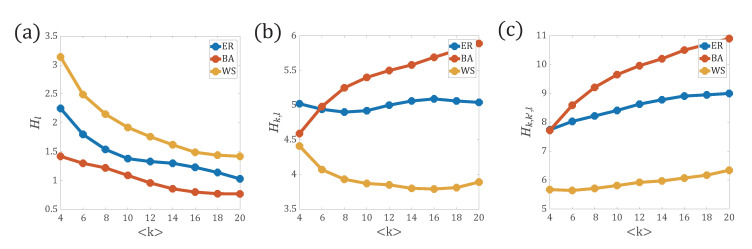
Comparison of network entropies defined on different path distributions on ER networks, Watts–Strogatz small-world (WS) networks, and Barabasi–Albert (BA) networks, respectively. (**a**) Network entropy $H_{l}$ versus average degree 〈k〉; (**b**) Network entropy $H_{k,l}$ versus average degree 〈k〉; (**c**) Network entropy $H_{k,k^{\prime },l}$ versus average degree 〈k〉. The parameters r=0.2 for WS networks.

In Figure 4a, we see that with the increase of the average degree 〈k〉, $H_{l}$ for the all three types of networks decreases monotonically. This result can be explained as, with the increase of the average degree 〈k〉, the network becomes denser and the distance between an arbitrary pair of nodes becomes less.

Hence, the path p(l) is narrowly distributed. For WS networks, since the paths are heterogeneously distributed, the entropy $H_{l}$ is the largest. For BA networks, since the distance between peripheral nodes and hub nodes is small, and the peripheral nodes can reach the remaining nodes through hub nodes, paths in BA networks are more narrowly distributed, thus, the entropy $H_{l}$ calculated with p(l) for BA networks is the smallest. The entropy $H_{l}$ for ER networks is in between the WS and BA networks.

In Figure 4b, the network entropies $H_{k,l}$ calculated with the path distribution with one end node’s degree, p(k,l), are compared for ER networks, BA networks, WS networks, respectively. For BA networks, with the increase of the average degree 〈k〉, since the length of paths between nodes is shorter, the path distribution becomes narrower, whereas the possible maximum degree becomes larger. Due to the variable possibility of the end nodes’ degree along the path, the distribution p(k,l) becomes wider and the network entropy $H_{k,l}$ increases monotonically with 〈k〉. In ER networks, with the increase of the average degree, the wider degree distribution and the narrower path distribution seem to play an opposite role in affecting the network entropy $H_{k,l}$, thus we see a stable curve of $H_{k,l}$ independent of the average degree. For WS networks, with the increase of 〈k〉, since the degree distribution does not change much and the path distribution becomes narrower, the curve of $H_{k,l}$ shows a significant downward trend.

Next, we explore how the network entropy $H_{k,k^{\prime },l}$ evolves with more information obtained from the two end nodes along the path for three types of networks in Figure 4c. With the increase of the average degree 〈k〉, the distance between nodes is shorter and the end nodes’ degree are increased, which results in a narrower path distribution and a broader degree values. Due to the limited possible values of path lengths, the end nodes’ degree plays a more fundamental role in evaluating $H_{k,k^{{}^{\prime }},l}$. Thus, $H_{k,k^{\prime },l}$ increases with 〈k〉 monotonically. Compared with the other two models, that is, WS networks and ER networks, due to the more heterogeneous degree distribution in BA networks, the network entropy value is the largest.

As shown by all the above experiments, network entropies, based on different probability distributions, can characterize the network structure in a certain extent. As more topological information is considered, the absolute value of the network entropy is also increased and the gap of the network entropy value between different types of networks is enlarged, which helps to discriminate networks more precisely.

#### 3.1.3. Comparison of Network Distance Based on Different Path Distributions

**Figure 5:**
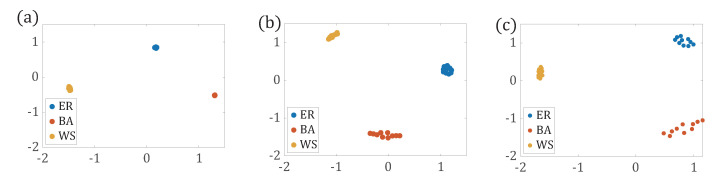
The Multi-dimensional Scaling (MDS) projections of the ER (blue points), BA (red points), and WS networks (orange points). (**a**) Projection of distance matrix $\sqrt{D_{JS}}$ between networks calculated with p(l); (**b**) Projection of distance matrix $\sqrt{D_{JS}}$ between networks calculated with p(k,l). (**c**) Projection of distance matrix $\sqrt{D_{JS}}$ between networks calculated with $p(k,k^{{}^{\prime }},l)$.

In order to understand if the three types of network models are discriminated by the proposed method, we generate 30 networks for each type of the network models, including ER model, WS model, and BA model. Then, we calculate the network distance between each pair of networks based on the path distribution, p(l), p(k,l), and $p(k,k^{\prime },l)$, respectively, and mapped it into the coordinate system by Multi-dimensional Scaling (MDS) method [34]. As shown in Figure 5, three types of networks are clearly detected as three clusters for all the three path distributions p(l), combined with one end node’s degree p(k,l), two end nodes’ degree $p(k,k^{{}^{\prime }},l)$. Furthermore, for each type of networks, with the introduction of the end node’s degree, the dissimilarity between networks is also amplified. More information on the end node’s degree is combined, the better the networks are separated.

### 3.2. Application of Network Comparison to Network Reduction in Multilayer Networks

Distance-based network comparison approach can also be applied to the issue of network reduction [7,20,35]. Network reduction aims to reduce the number of network layers to a minimum number by merging similar network layers while preserving more information of the multilayer networks. Multilayer networks are usually used to represent complex systems with multiple interactions among units and edges in one network layer represent one type of interaction. Given a multilayer network *G*, with *N* nodes and a total number of *M* network layers, the adjacency matrix of each subnetwork $G_{i}$ is represented as $A^{i}$ for i=1,⋯,M [36]. The reduction process of the multilayer network *G* is described as follows:

Step 1: Compute the topological distribution, $p_{i}$, for each subnetwork $G_{i}$. Then compute the distance between each pair of subnetworks $G_{i}$ and $G_{j}$, denoted as $d_{ij}$ and calculated by $d_{ij}=\sqrt{D_{JS}(p_{i}\left|\right|p_{j})}$.
Step 2: Calculate the average distance $D_{ave}$ between all pairs of subnetworks as the objective function, given by,
  (14) $$D_{ave}=\frac{2}{X(X-1)}\sum_{j=i+1}^{X}\sum_{i=1}^{X}d_{ij},$$
  where *X* is the number of subnetworks. If X=1, let $D_{ave}=0$ and stop the reduction process.
Step 3: Perform hierarchical clustering of layered networks. Aggregate subnetworks $G_{x}$ and $G_{y}$, whose distance $d_{xy}$ is the minimum, into a new subnetwork $G_{z}$. The updated adjacency matrix of the subnetwork $G_{z}$, $A^{z}$ is described as $A_{ij}^{z}=\max\left(A_{ij}^{x},A_{ij}^{y}\right)$, that is, edges in $G_{z}$ are the union set of the edges in $G_{x}$ and $G_{y}$.
Step 4: Update the multilayer network *G*, $G=G-\left\{G_{x},G_{y}\right\}\cup \left\{G_{z}\right\}$, that is, removing $G_{x}$ and $G_{y}$ from *G*, and adding $G_{z}$ to *G*. Then go to Step 1.

It is to note that the distribution $p_{i}$ can be arbitrary topological distribution of subnetwork $G_{i}$. In order to study the influence of different topological distributions in the reduction process, all the three path distributions, p(l),p(k,l) and $p(k,k^{{}^{\prime }},l)$, are applied in the multilayer structural reducibility procedure. Moreover, we choose the partition of the networks that maximizes $D_{ave}$ as the optimal structure of layered networks, that is, the distinguishability between the reduced network layers is as much as possible.

#### 3.2.1. Network Reduction on Synthetic Networks

In order to verify if the proposed method could distinguish three types of network models, we generate benchmark multilayer networks composed of three types of network models, each of which is generated with the same model but different connection by rewiring. For each MODEL∈{ER,WS,BA}, we generate MODEL0, that is, ER0,BA0,WS0, as basin networks, and then we randomly rewire n% (n∈{10,20,⋯,90}) of edges in MODEL0 to generate more networks with the same model. By doing so, networks generated with the same model are characterized by a different amount of edge redundancy. Totally, we have 30 subnetworks as layered networks, each of which is labeled as MODEL+n, where MODEL is one of {ER,WS,BA} and *n* is the percentage of rewired edges. Intuitively, networks generated with the same network model should be more similar than those generated with distinct mechanisms. For example, ER random networks should be more similar than BA networks. In the following, without specification, parameters are set as N=200 and the average degree 〈k〉=10. In the WS model, the rewiring probability *p* is set as p=0.2.

**Figure 6:**
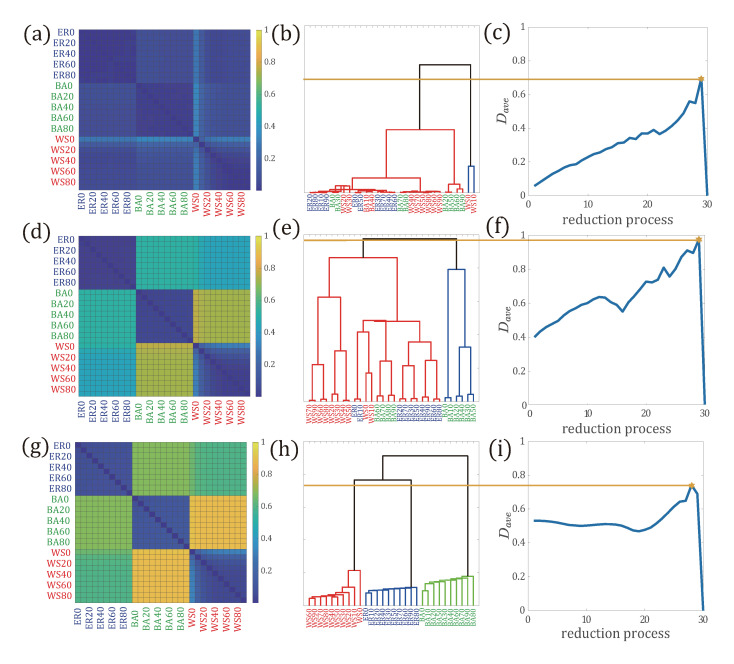
Network reduction process with different path distributions in multilayer networks generated with ER, WS, and BA networks. (**a**,**d**,**g**) (the **left panels**): The distance matrix between 30 layered networks calculated with the path distribution p(l), the path distribution with one end node’s degree p(k,l), and the path distribution with two end nodes’ degree $p(k,k^{\prime },l)$, respectively; (**b**,**e**,**h**) (the **middle panels**): The dendrogram of clustering process with different path distributions; (**c**,**f**,**i**) (the **right panels**): The average distance of layered networks $D_{ave}$ during the reduction process. The optimal reduced network structure is the one that corresponds to the maximal $D_{ave}$ (yellow star in the **right panels**).

In Figure 6a,d,g, we show the distance matrices calculated with the path distribution p(l), the path distribution with one end node’s degree, p(k,l), and the path distribution with two end nodes’ degree, $p(k,k^{\prime },l)$, respectively. We see that with the path distribution p(l), the three types of networks are not clearly separated (Figure 6a) due to the less difference in the distance measure. With more information being involved in the path distribution (Figure 6d,g), the distance between three types of networks is further amplified and the dissimilarity between them is enlarged. Distances between networks within the same group are much less than those in different groups, forming block matrices. Finally, distance calculated with $p(k,k^{{}^{\prime }},l)$ distinguishes networks of different groups best.

In addition, we can also aggregate networks in dendrogram according to the distance between networks as shown in Figure 6b,e,h. The final partition of the optimal reduced network is determined by the maximal distance $D_{ave}$ calculated during the aggregation process (Figure 6c,f,i). As expected, the reduction process based on p(l) aggregates networks randomly, and the distance between networks increases monotonically and then decreases to zero when aggregating as one network, as shown in Figure 6b,c.

For the dendrogram with the distribution p(k,l), we see that BA networks are partially partitioned as one group and the remaining networks as the other group (Figure 6e). Interestingly, by using the path distribution with two end nodes’ degree $p(k,k^{\prime },l)$, networks associated with the same network model, which are highly overlapped and similar, tend to be aggregated earlier (Figure 6h) and are clearly partitioned into three groups.

Therefore, incorporating more information on the end node’s degree into the path distribution is able to characterize network topology, and discriminate a network from another, especially in measuring dissimilarity between networks in network comparison.

#### 3.2.2. Network Reduction on Real Data

In this part, we testify whether our methods can detect typical variation in system status in temporal networks. We use the subset of interaction data from the Copenhagen network study, referred as Copenhagen Bluetooth data. Copenhagen Bluetooth data was recorded from university students over four weeks. We specify the duration of the time window, τ as 1 day, to partition the temporal network into 28 snapshots. Thus, the set of weekends is represented as $S_{weekend}=\left\{1,7,8,14,15,21,22,28\right\}$ and the set of the remaining weekdays is denoted as $S_{weekday}$. The network size is N=706. The multilayer network is built by taking each snapshot as one network layer and the total network layer is 28. Then, we implement network reduction process on the multilayer networks we built, and testify if our method can discriminate weekdays from weekends in the data set.

**Figure 7:**
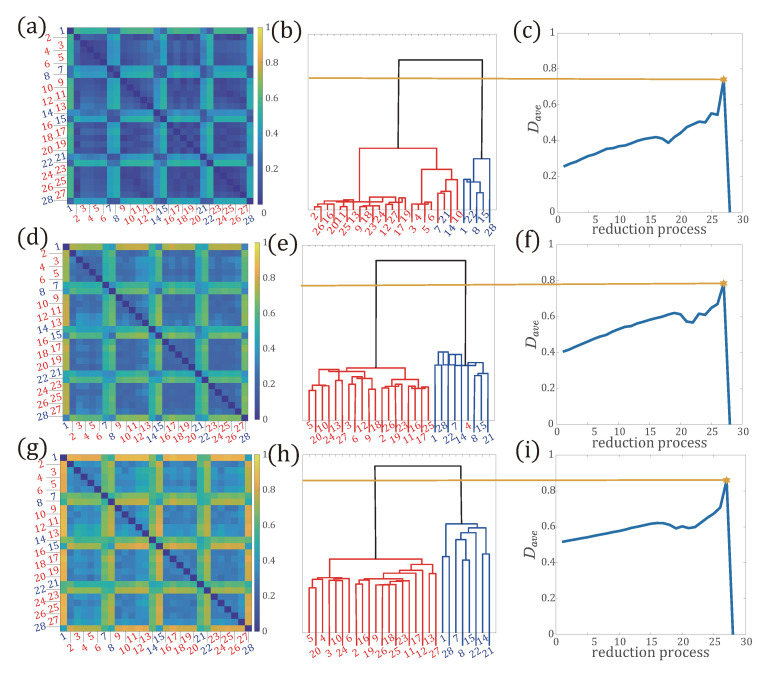
Network reduction process in multilayer networks built by the Copenhagen Bluetooth data. (**a**–**c**) (the **top panels**) are the reduction process based on p(l); (**d**–**f**) (the **middle panels**) are based on p(k,l); (**g**–**i**) (the **bottom panels**) are based on $p(k,k^{{}^{\prime }},l)$. (**a**,**d**,**g**): The distance matrices between layered networks; (**b**,**e**,**h**): The hierarchical clustering process; (**c**,**f**,**i**): The average distance $D_{ave}$ calculated during the reduction process. The optimal reduced network structure is the one that corresponds to the maximal value of $D_{ave}$ (yellow star in the **right panels**).

We calculate the distance between networks in days and then clustering networks in days according to the distance between them. In Figure 7a,d,g, we show the distance matrices obtained with distributions p(l) (a), p(k,l) (d), and $p(k,k^{\prime },l)$ (g), respectively. Firstly, we find that for all the distance matrices, a clear separation between weekdays and weekends is observed. The distance matrix suggests that the weekdays and weekends constitute distinct, in that the distance between weekdays or weekends in the same set is small, while it is large in different sets. With more information of the end nodes’ degree along the path, the distance between networks is enlarged (Figure 7b,c).

Secondly, observing Figure 7b,e,h, we see that with the three measures, the optimal reduced network is clustered into two groups (cut by yellow lines). During the clustering process with $p(k,k^{{}^{\prime }},l)$, we see that networks for weekdays cluster hierarchically, while networks for weekends cluster together (Figure 7h,i). In addition, it is to note that the reduction process based on p(k,l) mistakenly assigned weekday 4 to the set $S_{weekend}$ (Figure 7e). The performance of the reduction process based on p(l) is much unsatisfactory, since it mistakenly assigned weekends {7,14,21} to the set of $S_{weekday}$. Hence, from the above results, we conclude that incorporating more information on the end nodes’ degree along a path is able to capture characteristics of the network structure and measure the dissimilarity of the two networks.

## 4. Conclusions and Discussion

In this paper, we have introduced a path-based distribution measures by combining the end nodes’ centrality for network comparison, and validated its performance on synthetic networks and real data. The results reveal that the network entropy can effectively identify the tipping point in the process of network evolution, and the network distance can capture tiny difference between networks. These results also confirm our intuition that, more information on the end nodes’ degree of a given path is able to precisely quantify and amplify the structural difference between networks. As more nodes’ centrality is introduced to the path-based distributions, the paths are divided into more details. As a result, the distance between networks, that is, the difference between paths, increases. The application of the proposed measure in real world data also reveals that our method can efficiently identify the optimal network structure for multilayer networks and detect typical states in temporal networks.

It has to note that the proposed measure still has some limitations, such as higher computational complexity, since all the shortest paths between an arbitrary pair of nodes have to be computed. Hence, all the experimental results are simulated with relatively small network size. Moreover, we only take node’s degree as an example being introduced into path-based distribution, other node’s centrality measures such as betweenness can also be applied. As pointed by Meghanathan et al., there are poor correlations between degree-based centrality metrics (degree and eigenvector centrality) and the shortest-path based centrality metrics (betweenness, closeness, farness and eccentricity) for regular networks, but high correlations for scale-free networks [27]. Applying these centrality metrics into the path distribution to network comparison is still an opening question and can be explored in the future. The application of our proposed measure in network reduction of multilayer networks and identification of typical system status in temporal networks reveals the effectivity of our method. Further applications to other fields for network comparison are also expected in the future.

## Figures and Tables

**Table 1:** The path-based entropies of networks in the example in Figure 1.

	*G*	$G_{1}$	$G_{2}$	$G_{3}$	$G_{4}$
$H_{l}$	2.763	2.764	2.785	2.832	2.710
$H_{k,l}$	4.221	4.254	4.247	4.281	3.994
$H_{k,k^{\prime },l}$	5.025	5.199	5.166	5.175	4.487
